# Lab Retriever: a software tool for calculating likelihood ratios incorporating a probability of drop-out for forensic DNA profiles

**DOI:** 10.1186/s12859-015-0740-8

**Published:** 2015-09-18

**Authors:** Keith Inman, Norah Rudin, Ken Cheng, Chris Robinson, Adam Kirschner, Luke Inman-Semerau, Kirk E. Lohmueller

**Affiliations:** 1 Department of Criminal Justice Administration, California State University, East Bay, 25800 Carlos Bee Boulevard, Hayward, CA 94542 USA; 2 650 Castro Street, Suite 120-404, Mountain View, CA 94041 USA; 3 1224 Burnham Dr, San Jose, CA 95132 USA; 4 2854 Marsh St, Los Angeles, CA 90039 USA; 5 401 East 86th St., Apt 12H, New York, NY 10028 USA; 6 604 Lochmoor Ct, Danville, CA 94526 USA; 7 Department of Ecology and Evolutionary Biology, University of California, Los Angeles, 621 Charles E. Young Dr. South, Los Angeles, CA 90095-1606 USA

**Keywords:** Likelihood ratio, Forensic DNA, Probabilistic, Drop-out

## Abstract

**Background:**

Technological advances have enabled the analysis of very small amounts of DNA in forensic cases. However, the DNA profiles from such evidence are frequently incomplete and can contain contributions from multiple individuals. The complexity of such samples confounds the assessment of the statistical weight of such evidence. One approach to account for this uncertainty is to use a likelihood ratio framework to compare the probability of the evidence profile under different scenarios. While researchers favor the likelihood ratio framework, few open-source software solutions with a graphical user interface implementing these calculations are available for practicing forensic scientists.

**Results:**

To address this need, we developed *Lab Retriever*, an open-source, freely available program that forensic scientists can use to calculate likelihood ratios for complex DNA profiles. *Lab Retriever* adds a graphical user interface, written primarily in JavaScript, on top of a C++ implementation of the previously published R code of Balding. We redesigned parts of the original Balding algorithm to improve computational speed. In addition to incorporating a probability of allelic drop-out and other critical parameters, *Lab Retriever* computes likelihood ratios for hypotheses that can include up to four unknown contributors to a mixed sample. These computations are completed nearly instantaneously on a modern PC or Mac computer.

**Conclusions:**

*Lab Retriever* provides a practical software solution to forensic scientists who wish to assess the statistical weight of evidence for complex DNA profiles. Executable versions of the program are freely available for Mac OSX and Windows operating systems.

**Electronic supplementary material:**

The online version of this article (doi:10.1186/s12859-015-0740-8) contains supplementary material, which is available to authorized users.

## Background

The technology used to produce forensic DNA profiles has outpaced the tools available to interpret and provide statistical weight to the results. The chemistry, software and hardware used in forensic DNA typing has, over the relatively short life of the discipline, become both highly sophisticated and extremely sensitive. In addition, laboratories receive and analyze samples derived from contact or “touch” DNA, resulting in DNA profiles which cannot be traced to a visible biological deposit or a specific physiological fluid. These samples frequently contain contributions from multiple donors, and may be of poor quality and low quantity, resulting in complex profiles [1].

One particular challenge associated with such complex samples is allelic drop-out. Drop-out refers to the situation where one or more alleles from a contributor to a DNA sample is not detected in the DNA profile [1]. Drop-out creates a discordance between the genotype of the true contributor and the DNA profile detected in the evidence.

Forensic DNA laboratories have historically struggled to provide appropriate statistical weights for such complex profiles. Until recently, especially in the US, simple statistical methods, such as the Random Match Probability (RMP), restricted RMP, the Combined Probability of Inclusion (CPI), or restricted CPI have been applied only to those genetic loci where the peak heights in the DNA profile for the evidence sample suggest drop-out may be unlikely, and omitting from the calculation those loci where dropout is likely [2, 3]. Such an approach has been employed in an attempt to provide a “conservative” estimate of the statistical strength of complex profiles (i.e. they underestimate the strength of the evidence supporting the presence of a particular contributor). However, such a framework encompasses the dual risk of underestimating the strength of the evidence with respect to a potential contributor of interest or, alternatively, falsely including a potential contributor [4, 5]. Although, to address these issues, the CPI approach has been extended to explicitly and more properly model the possibility of allelic drop-out [6, 7], these extended methods have not, to our knowledge, been widely adopted.

In contrast, probabilistic approaches using a likelihood ratio (LR) framework are widely accepted to solve problems in systems where the data are continuous and may contain ambiguity [4, 8–13]. Specifically, the LR is the ratio of the probability of the evidence (i.e. the DNA typing results) under one hypothesis to the probability of the evidence under a second hypothesis. For example, if an allele from the suspected contributor is not detected in the evidence profile, one hypothesis is that the suspected contributor was indeed a contributor to the evidence profile, but his allele was not detected because it dropped out. A second hypothesis is that the suspected contributor’s allele was not detected in the evidence profile because he did not actually contribute to the profile. Instead, a random unknown individual would be the source of the profile. The LR is the ratio of the probability of the evidence under each of these scenarios. For example,

$$ LR=\frac{P\left(E\Big|\mathrm{Suspect}\ \mathrm{is}\ \mathrm{the}\ \mathrm{source}\ \mathrm{of}\ \mathrm{the}\ \mathrm{evidence}\ \mathrm{profile}\right)}{P\left(E\Big|\mathrm{Random}\ \mathrm{person}\ \mathrm{is}\ \mathrm{the}\ \mathrm{source}\ \mathrm{of}\ \mathrm{the}\ \mathrm{evidence}\ \mathrm{profile}\right)}. $$

One hypothesis is considered in the numerator of the LR and the other is considered in the denominator. The LR framework allows one to explicitly compare the probability of the evidence under both of these scenarios. One potential disadvantage of the LR approach is that one is required to specify the number of contributors, which may not be known with certainty. While the LR approach has enjoyed extensive theoretical support for dealing with complex forensic DNA profiles [4, 8–13], one impediment to the widespread adoption of it by forensic laboratories has been a lack of freely available, user-friendly software to perform such calculations. The software detailed here, *Lab Retriever*, automates calculations to perform a LR that incorporates a probability of drop-out (*P*(*D*_*O*_)). Ultimately, the LR results are used to infer the strength of the evidence in support of one proposition relative to another regarding who contributed to the DNA evidence profile.

## Implementation

*Lab Retriever* derives from an open-source R-code program introduced by David Balding [4]. On the front end we created a graphical user interface (GUI) coded using a combination of JavaScript, css, html and Python to improve the user experience for the typical forensic DNA analyst who is not a computer programmer. On the back end, we re-coded the program using C++ to increase the run speed.

First we describe the general form of the LR computed by *Lab Retriever*. Consider the following hypotheses:

H0: The suspect is the source of the evidence profile.

H1: A random individual is the source of the evidence profile.

Then the LR computed by *Lab Retriever* can be written as:

$$ LR=\frac{P\left(E\Big|s\right)}{{\displaystyle \sum_{\mathrm{all}\ j}P\left(E\Big|j\right)P(j)}}. $$

Here *P*(*E*|*s*) is the probability of seeing the evidence profile if the suspected contributor, *s*, is the source. It is computed considering the probabilities of the particular drop-out and drop-in events required to convert the *s* profile into the profile seen in the evidence, *E*. The denominator is the probability of the evidence if a random individual is the source. This can easily be computed using the law of total probability, conditioning on each possible genotype *j* at the locus and summing over all possible genotypes. For a particular genotype *j*, we compute *P*(*E*|*j*) the same way as in the numerator. This is the probability of the necessary drop-out and drop-in events required to convert genotype *j* into the evidence profile, *E. P*(*j*) is the probability of sampling genotype *j* from the population and is computed using population genetic models as described below. This LR can be extended to consider multiple unknown contributors by summing over sets of genotypes. Further details of the algorithms are provided in Balding and Buckleton [4]. In *Lab Retriever*, rather than iterating over all possible genotypes, which can become slow when considering multiple unknown contributors, we use a dynamic programming algorithm to calculate this probability in the denominator (Additional file 1), resulting in computational speedups.

We modified certain population genetic and statistical models. The original program of Balding and Buckleton [4] uses a pseudo-count approach, which assumes that the allele frequencies follow a Dirichlet distribution, to account for uncertainty in the estimates of the population allele frequencies. Essentially this procedure is equivalent to adding the alleles seen in the evidence profile and the alleles seen in the suspected contributor’s reference profile to the population database of allele frequencies. Because this approach is not commonly used in US laboratories, *Lab Retriever* uses an alternate procedure that sets any allele in the suspected contributor or evidence profile whose frequency is <5/2*n* to have a frequency of 5/2*n* [12, 14] where *n* is the number of individuals in the allele frequency database.

However, *Lab Retriever* does use the coancestry adjustment (*θ*, or F_ST_) suggested in the original program of Balding [4]. The purpose of this adjustment is to account for the possibility of distant (population-level) relatedness between the suspected contributor and the true contributor. For ease and speed of computation, in both the original R code [4] and in *Lab Retriever*, the co-ancestry adjustment is implemented at the allelic level, rather than performed on genotypes. For example, if the hypothesized contributor is homozygous for the A allele, we compute the conditional probability of sampling an additional copy of the A allele given that the suspect has the AA genotype *P*(A|AA). This essentially uses the Balding-Nichols sampling formula [15], but for sampling a single allele rather than a complete genotype. Examination of example profiles indicates that this approach is intermediate in effect between the population structure adjustments made in NRC II equations 4.4 and 4.10. (Lohmueller, Rudin, and Inman, in preparation).

In *Lab Retriever*, *P*(*D*_*O*_) refers to the probability that an allele at a locus drops out [4]. Assuming that alleles drop out independently of one another, the nominal probability that a homozygous genotype (two of the same) would drop out is *P*(*D*_*O*_)^2^. However, to account for the observation that complete drop-out of a homozygous genotype occurs at a frequency somewhat less than simply the square of the drop-out frequency of a single allele, the term is modified by *α* such that the probability of a homozygote dropping out would be *αP(D*_*O*_ *)* ^2^. We use *α* = 0.5 as suggested by Balding and Buckleton [4] A similar approach is used to compute the probability that an allele carried by multiple contributors has dropped out. Specifically, for *n* copies of an allele, the probability of that allele dropping out is computed as *α* ^*n-1*^ *P(D*_*O*_ *)* ^n^.

Executable binaries are available for both Mac OS X and Windows. The program runs on any standard modern computer.

## Results and discussion

### Overview of the program

**Fig. 1:**
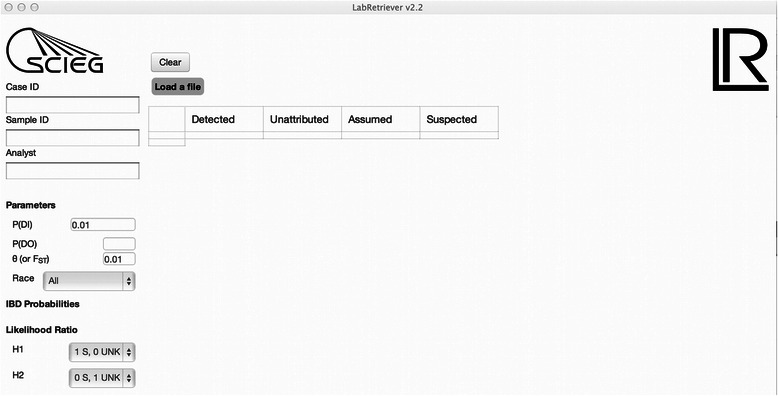
Input screen for *Lab Retriever*. The text contains a detailed description of the input parameters

*Lab Retriever* will calculate LRs comparing the probability of the evidence under different hypotheses, while allowing for allelic drop-out. The user must specify as input the alleles that were detected in the evidence profile, the genotype of the suspected contributor who is being compared to the evidence profile, the genotypes of any assumed contributors, the specific hypotheses to consider, and the allele frequency databases to use. Additionally, the user must supply values for the following parameters: the probability of drop-out, the probability of drop-in, and the value of the co-ancestry adjustment. Figure 1 depicts the *Lab Retriever* input screen. Below we describe in detail the different kinds of input for the program.

### Preparation of data

*Lab Retriever* can accept output files from any genetic analysis software capable of exporting data in a non-proprietary spreadsheet format. The data are typically exported from such software directly as a .csv or .txt file, but data can also subsequently be saved in one of these formats if originally exported in a different format. At this time, all of the commonly used genetic analysis programs of which we are aware, including GeneMapper® ID, GeneMapper® ID-X, OSIRIS, and GeneMarker® readily export data in these file formats. Data files can also be modified, or even created *de novo,* by the user. *Lab Retriever* requires, at a minimum, the sample name, the genetic locus designation (marker), and the genetic types (alleles) to be included in the exported file. The column headers recognized by the program are: “sample file” or “sample name” to designate each sample containing genetic data, “marker” to designate the genetic locations (loci) typed in the sample set, and “allele 1,2,3…x” to designate the genetic variants detected at each locus.

It is recommended that the “peak height” of each allele also be exported. While *Lab Retriever* does not directly use peak heights, which represent the relative amount or mass of DNA, it is recommended that the user employ this data outside of the program to determine an empirical *P*(*D*_*O*_). This parameter will be described more fully in the next section. As an adjunct to *Lab Retriever*, we provide a “universal drop-out calculator” that can be employed by the user to estimate *P*(*D*_*O*_) for any particular sample. This calculator, provided in the form of an Excel spreadsheet, incorporates previously determined logistic regression parameters to predict *P*(*D*_*O*_) for the evidence sample as a function of the average heights of the detected peaks [16]. These parameters were estimated in previous work using samples with known amounts of drop-out (Table 2 of Lohmueller et al. [17]). Further details of the estimation of drop-out probabilities may be found in [5, 17]. Users are encouraged to use their own validation data to estimate the parameters of the logistic regression model that are specific for their own typing system and laboratory.

Although not requisite to the program *per se*, we strongly recommend that the user determine and apply an empirical analytical threshold (AT) to any evidence DNA profile destined for statistical analysis using *Lab Retriever*. The AT is the peak height above which the user can be confident that a peak is a real allele, rather than instrument noise [18–23]. Setting an empirical AT optimizes data capture, thus providing the program with maximal data input. Any number of different approaches may be employed to determine an empirical AT; we recommend using negative control samples to determine either a “limit of quantitation” (mean of instrument noise plus 10 standard deviations (sd) or a proxy (2× maximum instrument noise) calculated by measuring the highest noise peak and multiplying by 2). An AT is not intended to avoid artifacts such as sporadic electrical spikes, incomplete color separation (“pull-up”), or chemical impurities (“blobs”) [18–23]. An empirical AT can be determined either on a run-specific basis or using cumulative historical data. In addition, because the fluorescent dyes attached to the DNA fragments as part of the detection scheme exhibit differential sensitivity, maximizing allele detection is realized by determining a separate AT for each dye channel.

After the data are properly analyzed and exported, the .csv data file is imported into *Lab Retriever*.

### Program parameters

*Lab Retriever* can currently accommodate autosomal STR data comprising the genetic marker sets typed by the Globalfiler® and PowerPlex® Fusion genetic analysis kits. These kits represent the most current technology in use by forensic DNA laboratories. Additional genetic markers can readily be added to the look-up files containing the population frequency data. Previous genetic marker sets comprise various subsets of this current group and thus can readily be accommodated. The program automatically detects the marker set present in the imported data of the evidence sample and only presents results for markers present in the “Detected” profile chosen from the imported file.

*Lab Retriever* currently includes Caucasian, African-American, and Hispanic population data from which the allele frequencies used in the calculations are derived. The population data were collected from individuals in the US and made available by the National Institutes of Standards and Technology (NIST) and are available from the STRbase web site as the NIST 1036 US population dataset [24].

The *P*(*D*_*O*_) parameter is key to the utility of *Lab Retriever*. An estimate of the probability that the DNA profile might be missing information due to low levels of input DNA, either *in toto*, or per contributor, is key to properly weighting the evidence for or against the proposition that any particular individual has contributed DNA to the profile. The empirical estimate, derived as explained in the previous section, is input in the field marked *P*(*D*_*O*_). The user is encouraged to estimate this parameter for a given sample using a system and lab-specific logistic regression model. It can also be useful to calculate LRs using a range of *P*(*D*_*O*_)s to examine the effect of different values. This can be accomplished by sequentially replacing the value in the *P*(*D*_*O*_) field and re-running the calculation. In the current implementation, *P*(*D*_*O*_) is assumed to be the same across all loci. While this omits some real biological complexity, we previously found that LRs computed using this drop-out probability were similar to those obtained when using a benchmark drop-out probability that varied across loci (see [17] for further details). Further, for profiles with multiple contributors, the same *P*(*D*_*O*_) is used for all contributors.

The probability of drop-in *P*(*D*_*I*_) is the probability that exactly one allele will drop-in at a locus. It is essentially a nuisance parameter included to take into account low levels of laboratory contamination that might be detected, usually using certain analytical enhancements that increase the sensitivity of the analysis [4, 25]. We use the model of drop-in as described by Balding and Buckleton [5] (Additional file 2). A default value of 0.01 automatically populates the input *P*(*D*_*I*_) field. This would be considered a relatively large value, as a laboratory experiencing a 1 % frequency of contamination per locus would be alerted to a greater problem. Conversely, a *P*(*D*_*I*_) value of 0.01 makes very little difference in the final LR result. An empirical estimate of *P*(*D*_*I*_) can be determined for any particular laboratory situation by examining cumulative historical data on negative control samples.

*Lab Retriever* allows the user to propose hypotheses about relatives [4, 15] by accounting for alleles identical by descent (IBD) shared between the suspected contributor and a relative. Typically, the numerator hypothesis specifies the presence of a suspected contributor. In the denominator the suspected contributor is typically replaced by an unknown individual who is unrelated to the suspected contributor. *Lab Retriever* also allows one to calculate a LR in which one of the unknown contributors in the denominator represents a random specified relative rather than a random unrelated individual. For example, one can compute the following LR:

$$ LR=\frac{P\left(E\Big|\mathrm{Suspect}\ \mathrm{is}\ \mathrm{the}\ \mathrm{s}\mathrm{ource}\ \mathrm{of}\ \mathrm{the}\ \mathrm{evidence}\ \mathrm{profile}\right)}{P\left(E\Big|\mathrm{Suspect}\hbox{'}\mathrm{s}\ \mathrm{brother}\ \mathrm{is}\ \mathrm{the}\ \mathrm{s}\mathrm{ource}\ \mathrm{of}\ \mathrm{the}\ \mathrm{evidence}\ \mathrm{profile}\right)}. $$

A drop-down field within the main program window allows the user to fill in the appropriate probability that the relative shares 0, 1, or 2 alleles IBD with the suspected contributor. These probabilities depend on the specific relationship between the two individuals (e.g. siblings, parent–child, etc.) and follow from genetic first principles. Charts of basic relationships can easily be found in the literature, and are also included in the *Lab Retriever* user manual. The IBD option is not required or appropriate when the profiles of the related individuals are known.

The main program window contains two drop-down fields in which hypotheses for the numerator and denominator of the LR can be specified separately (Fig. 1). Currently *Lab Retriever* can handle profiles with up to 4 unknown contributors. Therefore, in the denominator, selections can be made for various combinations up to and including 4 unknowns; as at least one suspected contributor is typically named for the numerator, selections can be made that include combinations of up to 3 unknowns. The various combinations of hypotheses available cover a wide spectrum of situations commonly encountered in forensic DNA laboratories.

Three categories of contributors comprise the total evidentiary profile within *Lab Retriever*: Suspected, Assumed, and Unknown (Fig. 1). Together these categories make up the total number of contributors in the profile. No upper limit exists for the number of assumed donors; the number of assumed donors is counted toward the total number of contributors assumed for the profile, but is not considered in either the numerator or denominator when picking the hypotheses. The model of drop-out does not apply for assumed donors. If an allele in the assumed donor is not found in the evidence profile, it should not be included in the detected input profile. The peak is not included because it cannot mask a minor allele if it is not detected in the profile.

By default, the co-ancestry coefficient, *F*_*ST*_ (also called θ) is set to 0.01 to reflect the value currently used in most US forensic laboratories. However, the user has the option to supply other values.

### Program output

**Fig. 2:**
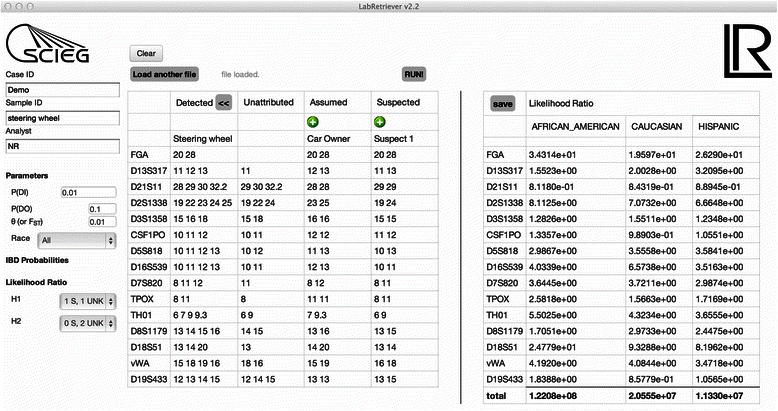
Output screen for *Lab Retriever*. The left side of the screen shows the alleles present in an example profile. The right side shows the final LRs computed for each locus, as well as the final LR computed across loci, using the three different population allele frequency databases

Figure 2 shows an example of the output of *Lab Retriever*. The results screen shows the overall LR for each of the chosen population groups. The LR for each individual locus is also displayed, allowing the user to examine, for example, a possible source of unexpected results. The input information and parameters, as well as the output results, can then be saved in a user-defined location. The saved file can be opened and edited in Excel or any other spreadsheet program that can open a .csv file.

### Comparison with existing software

While the lack of freely-available automated software has inhibited adoption of probabilistic approaches to forensic DNA genotyping, the past few years have seen the introduction of a number of software solutions aimed at this audience. These tools may be categorized according to various criteria, including analytical approach, computer requirements, and license requirement. Because this field is in flux and rapidly evolving, even a best attempt to provide a comprehensive listing would surely be out of date by the time this paper is published. However, a representative listing will be sufficient to provide a useful comparison of *Lab Retriever* to other available software. Table 1 details the salient attributes of a variety of current software products for probabilistic genotyping. Recent papers by Steele and Balding [26] and Cowell et al. [27] list additional information about some of these approaches and software implementations and we refer the interested reader to this work.

**Table 1:** A non-exhaustive list of software for assessing the weight of complex forensic DNA profiles

Name	Computer requirements	Licensing model
STRmix™ [37]	Windows only	Commercial, proprietary
True Allele® [13, 28]	Mac, Windows interface^c^	Commercial, proprietary
LRmix, LRmix Studio [29, 34, 38]	Mac, Windows, Linux	Open source, freely available
Lab Retriever	Mac, Windows	Open source, freely available
LikeLTD [30]	Mac, Windows, Linux	Open source, freely available
Armed Expert™^a^	Windows only	Commercial, proprietary
DNA View™	Windows only	Commercial, proprietary
DNA Mixtures	Mac, Windows	Open source^b^

^a^This program employs modules that provide options for various different approaches

^b^Dependent on a commercial program

^c^This program requires multiple computer processors; all other programs work on stand-alone single computers

### Capabilities and limitations

Three general categories of factors contribute to the complexity of a DNA profile: internal factors (biology and genetics), external factors (environment), and analytical factors (chemistry and instrumentation). As a consequence, both the quantity (template amount) and quality (DNA fragment size, chemical contamination, biological contamination) of any particular sample may be affected. The potential resulting DNA degradation, PCR inhibition, PCR sampling error (stochastic effects), or biological contamination, may variously impact the complexity of a DNA profile. As a result, the profile that is detected after DNA typing may be complicated by multiple contributors, incomplete information and artifactual elements, either biological or chemical.

Various publications have described the insufficiency of the historical binary statistical models in accommodating the ambiguity of these profiles. The most explicit was the 2006 ISFG Commission report [8] which described how failure to account for the possibility of allelic drop-out in the evidentiary sample could, in particular, overweight the evidence against a suspected contributor, typically a defendant. This publication warned the forensic community that it must move to probabilistic genotyping models that could model complex profiles, a caution that was underscored in the 2012 publication by the same group [9]. In the intervening years, a number of other publications accrued [4, 13, 26, 28–30], each accentuating the need to be able to both use more of the information content in a profile, (e.g. modeling of peak heights in multi-contributor profiles) to provide appropriate weight and also to account for missing information (allelic drop-out) or extraneous content (contamination).

One of the more recent publications [31] attempts to categorize various approaches in order to simplify the comparison for the forensic DNA community. Unfortunately, the categorization of probabilistic approaches into “semi-continuous” and “continuous” (sometimes referred to as “fully continuous”) is overly simplistic and presents a false dichotomy. A more transparent description of this distinction would be that some systems directly model all of the quantitative peak height information in a fully automated fashion, while others summarize the data by whether peaks are present, absent, or uncertain. Obviously the latter systems still allow for manual accommodation of peak heights to some extent prior to data entry into the program. However we would assert that differences across software systems go beyond whether peak height information is used; each approach uses a different underlying model and each encompasses, as well as emphasizes, a different subset of parameters. Table 2 lists an extensive, although likely non-exhaustive, set of factors that could contribute to the ambiguity of complex profiles.

**Table 2:** Factors affecting the complexity of DNA profiles

Peak heights	
Stutter	
Masking by a major peak	
Masking by a stutter peak	
Analytical threshold	
Number of contributors	
Probability of drop-out	
Probability of drop-in	
Population genetic model	
Population frequencies	
Consideration of close relatives	

Part of the challenge in creating an approach to assess the strength of a complex sample is choosing which of these aspects to model, and how to implement the model. None of the software offered to the forensic DNA community simultaneously considers all these factors. Thus the specifics of each approach, how the data are summarized, the parameters that are included in the model, and the particular implementation, form the basis for the differences between the various software tools that embrace a general probabilistic approach.

*Lab Retriever* incorporates the following information in calculating a LR: allele calls based on an empirical AT, an empirically-determined *P*(*D*_*O*_) for the relevant portion of the evidence profile, an (ideally) empirically-determined *P*(*D*_*I*_), published allele frequencies, and a user-determined value to adjust the allele frequencies for distant relatedness between the suspected contributor and the true contributor. *Lab Retriever* accommodates uncertainty due to potential allele masking of peaks in a minor profile by peaks from a major profile (either parent or stutter) by having the user define the potentially masking alleles as an “assumed” profile. *Lab Retriever* does not automatically incorporate peak height information, or any other parameter not specified above, in calculating an LR. Peak height information can, to a certain extent, be incorporated manually by the user during preparation of the input data file.

We are aware of only two formal studies comparing a few of the currently available programs. Both studies attempted only modest comparisons. In the first study, *Lab Retriever*, LRmix and STRmix™ were compared for a single source profile and for a simple mixture comprising a major and minor contributor [32]. Neither of the profiles was missing any information; therefore neither analytical threshold nor allelic drop-out influenced the results. All three programs produced similar results for the single source contributor, as well as for the major contributor to the mixture. As expected, STRmix™ produced larger absolute LRs for the true minor contributor to the mixture. *Lab Retriever* and LRmix produced similar, but smaller, LRs. Importantly, all three programs produced LRs strongly supporting the proposition that the true minor contributor was present in the mixture; thus leading to the same correct inference regarding the composition of the sample. The second study compared STRmix™ and *Lab Retriever*, as well as a couple of historical binary models, and also introduced mixed profiles with potential allelic drop-out [33]. This study also, unsurprisingly, demonstrated that, when relying solely on automation to model peak heights, STRmix™ produced larger LRs in profiles in which the ratios of the major and minor components show greater divergence. And again, like the previous study, both approaches strongly supported the proposition that the true minor contributor was present in the mixture; thus leading to the same correct inference. However, because *Lab Retriever* was not run as recommended 1 (two authors of [33] are architects of STRmix™, and none of the four authors had been formally trained on *Lab Retriever*), the results reported in this study do not reflect a full and complete comparison of these programs.

Insufficient work has been performed to date to completely understand the limits that distinguish the various models and implementations. One of the difficulties in comparing the different programs is that several are proprietary commercial products that are quite expensive. Thus it is unrealistic for any one person or entity to own all of the different products. Further, each relies on different assumptions and employs a different model; as such each requires very specific training on proper implementation. Again, it is difficult for any one person or entity to acquire proper training and be fully expert in the use of all the different software.

A special instance of comparison is that of an upper bound to a reported LR. Is a larger numerical statistic meaningful, or is it an artifact of the particular algorithms implemented within a piece of software? Cowell et al. have demonstrated that the upper limit for an LR conditioned on one suspected contributor is the random match probability (RMP) [27]. Thus testing a model should calibrate calculated LRs against this numerical limit, which reflects the theoretical upper bound for a complete single source sample. For complex profiles, the idea of calibrating a LR result against known non-contributors has gained traction as a more useful metric than a simple comparison of numerical magnitude [34–36]. Another important point to consider is that, in the milieu in which *Lab Retriever* is intended to be used, the absolute numerical value of the LR is a less important metric of utility than the ultimate inference to which it leads. This inference is informed by both the spread of LR results between the suspected contributor and known non-contributors, and the numerical value of the LR. For example, an LR of 10^10^ will lead to the same inference, and have similar consequences for the justice system as an LR of 10^12^ or 10^20^. In this example, all of these results strongly support the inference that the suspected contributor is the true contributor. Any policy decision by a laboratory to impose a convenience limit on reported results should be informed by research and validation. That work should ideally encompass both validation using samples for which ground truth is known, as well as research into how jurors perceive numerical magnitude. Thus, while different approaches may produce results of varying magnitudes, the decision about which software to adopt will include other factors, such as cost/benefit, ease of use, flexibility and support. It is expected that information about the relative capabilities of the various models and tools will emerge over the coming years.

### Intended use

*Lab Retriever* is primarily intended to assist with interpretation and weighting of forensic DNA evidential profiles.

### Future development

We intend to continue development of all of the components of *Lab Retriever*. Planned improvements include: user-defined α, user-defined population allele frequency databases for existing populations, addition of additional population allele frequency databases, built-in known non-contributor simulations, built-in *P*(*D*_*O*_) estimator, ability to analyze multiple replicates, and automation of sequential runs of multiple *P*(*D*_*O*_)s. We also propose to explore more advanced models of allelic drop-out, such as those previously proposed [13, 34] and those including locus-specific and contributor-specific drop-out probabilities.

## Conclusions

With *Lab Retriever*, we have provided the forensic DNA community with freely available open-source software to assist in interpreting and weighting evidentiary profiles. While other tools exist that may be more powerful, the cost/benefit relationship of the various tools has yet to be established. We contend that *Lab Retriever* can, especially with an optimized input data file, handle many or most of the complex profiles with which forensic DNA analysts currently struggle. Key features of *Lab Retriever* include its user-friendly graphical user interface, the ability to perform calculations with up to four unknown contributors nearly instantaneously, and, of course, democratic access to both the software package and underlying computer code.

## Availability and requirements

**Project name:** Lab Retriever

**Project home page:** http://scieg.org/lab_retriever.html; https://github.com/SCIEG/LabRetriever

**Operating system(s):** Windows 7 and above/Mac OSX Lion and above

**Programming language:** C++/Python/JavaScript

**Other requirements:** TideSDK (deprecated)

**License:** Creative Commons; http://creativecommons.org/licenses/by-nc-sa/4.0/legalcode

**Any restrictions to use by non-academics:** None

## Additional files

Additional file 1:
**Modification of the Balding algorithm.** (PDF 159 kb)
Additional file 2:
**Description of the drop-in model used by** ***Lab Retriever.*** (PDF 90 kb)

## Abbreviations

LR: Likelihood ratio
*P*(*D*_*O*_): Probability of drop-out
*P*(*D*_*I*_): Probability of drop-in
RFU: Relative fluorescent unit
CPI: Combined probability of inclusion
RMP: Random match probability
IBD: Identity by descent

## Footnotes

1 Several examples of errors include: 1) the profiles were analyzed at a convenience analytical threshold of 50 rfu, rather than at an empirically determined analytical threshold as recommended; 2) a recommended procedure to account for possible masking of minor alleles by peaks in the stutter position of major peaks was not employed; 3) Peak height differences were blindly ignored rather than employing the recommended procedure for manual deconvolution prior to running *Lab Retriever*. However, a manual deconvolution was employed for the RMP comparison in the same paper; 4) Major profiles were not assumed, as would typically be the procedure in casework. Because the raw electronic data were not provided as supplemental material, it was impossible to rerun the samples using the recommended guidelines to determine how the results might have differed.
