# Supplementary material for: Lab Retriever: a software tool for calculating likelihood ratios incorporating a probability of drop-out for forensic DNA profiles
Source: BMC Bioinformatics. 2015 Sep 18;16:298. doi: 10.1186/s12859-015-0740-8 (PMC4575494; doi:10.1186/s12859-015-0740-8)
Supplement: Additional file 1: — Modification of the Balding algorithm. (PDF 159 kb) [file 12859_2015_740_MOESM1_ESM.pdf]

Additional File 1: Modification of the Balding algorithm

Lab Retriever uses the model proposed in [1] to calculate the likelihood of low-template DNA evidence involving drop-out and drop-in probabilities. More specifically, given low-template DNA found at a crime scene (known as the crime scene profile, or CSP), the model calculates the probability of observing the low-template DNA profile if it was left by a particular person, taking into account of possible allelic drop-out or drop-in. Though it uses the same model, we have designed an algorithm to increase the computation speed of Balding's original program by many orders of magnitude. Computations that may have taken hours or days on some machines can now be performed in fractions of a second.

To understand the algorithmic acceleration, first we present a simplified version of Balding's model. This model lacks certain features, such as multiple loci, masked alleles, and sampling adjustment parameters (Fst/θ), to prevent the mathematics from becoming too complex. However, these can be incorporated into the model with relative ease.

For a locus ℓ, denote the set of all alleles of ℓ as A_ℓ, and denote the set of alleles of ℓ found in the CSP as CSP_ℓ. Denote the frequency of an allele a in a certain population as $p(a)$. Let the probability of drop-out for one copy of one allele be D_o. The probability of n copies of one allele dropping out is modeled by $\alpha^{n-1}D_o^n$ for some value α (which is determined experimentally to be approximately 0.5). Let the probability of drop-in of one allele be D_i. The probability of drop-in of exactly n different alleles is modeled by D_i^n for all $n > 1$. The probability of no drop-in for a locus ℓ is then

$$1 - D_i - D_i^2 - \ldots - D_i^{|A_\ell|} = \frac{1 - 2D_i + D_i^{|A_\ell|+1}}{1 - D_i}$$

Denote this value as $\bar{C}_\ell$. Note in this model, the drop-out and drop-in probabilities do not depend on the source of the allele (whether it is from a suspected contributor or an unknown individual).

Define a *count* function that takes a list of alleles T and an allele a, and returns the number of times a appears in T. Let $f_{\ell,n}$ be the family of functions

that takes a list T of $2n$ alleles of ℓ and calculates the following:

$$f_{\ell,n}(T) = g_\ell(T) \prod_{a \in A_\ell} \begin{cases} 1 - \alpha^{count(T,a)-1} D_o^{count(T,a)}, & \text{if } a \in T, a \in CSP_\ell \\ \alpha^{count(T,a)-1} D_o^{count(T,a)}, & \text{if } a \in T, a \notin CSP_\ell \\ p(a) D_i, & \text{if } a \notin T, a \in CSP_\ell \\ 1, & \text{if } a \notin T, a \notin CSP_\ell \end{cases}$$

where

$$g_\ell(T) = \begin{cases} \bar{C}_\ell, & \text{if } \forall a \in CSP_\ell, a \in T \\ 1, & \text{otherwise} \end{cases}$$

For convenience, the conditions of the piecewise functions above are interpreted here:

Condition	Meaning
$a \in T, a \in CSP_\ell$	No drop-out occurred for allele a
$a \in T, a \notin CSP_\ell$	Drop-out occurred for allele a
$a \notin T, a \in CSP_\ell$	Drop-in occurred for allele a
$a \notin T, a \notin CSP_\ell$	No drop-in occurred for allele a
$\forall a \in CSP_\ell, a \in T$	No drop-in occurred for all alleles in the CSP

Note that if it were possible to assign a person's alleles an ordering, $f_{\ell,n}$ calculates the likelihood that n people, with alleles (T_1, T_2), (T_3, T_4), and so on, were the only DNA contributors to the crime scene considering only the evidence for locus ℓ. Finally, we can calculate likelihoods of hypotheses involving unknowns and suspects using the following:

$$\text{1 unknown} = \sum_{a_1^1 \in A_\ell} \sum_{a_2^1 \in A_\ell} p(a_1^1) p(a_2^1) f_{\ell,1}([a_1^1, a_2^1])$$

$$\text{2 unknowns} = \sum_{a_1^1 \in A_\ell} \sum_{a_2^1 \in A_\ell} \sum_{a_1^2 \in A_\ell} \sum_{a_2^2 \in A_\ell} p(a_1^1) p(a_2^1) p(a_1^2) p(a_2^2) f_{\ell,2}([a_1^1, a_2^1, a_1^2, a_2^2])$$

$$\text{1 suspect with alleles } a \text{ and } b = f_{\ell,1}([a, b])$$

$$\text{1 suspect with alleles } a \text{ and } b\text{, 1 unknown} = \sum_{a_1^1 \in A_\ell} \sum_{a_2^1 \in A_\ell} p(a_1^1) p(a_2^1) f_{\ell,2}([a, b, a_1^1, a_2^1])$$

Note that since ordering matters, the case where a person has alleles (a, b) is different from the case where a person has alleles (b, a); this is practically impossible to distinguish, but since the (a, b) and (b, a) cases are twice as common as the (a, a) cases, the mathematics works itself out. (This corresponds to a heterozygote's genotype frequency of $2pq$ versus a homozygote's genotype frequency of p^2.) Also note that the suspect cases are simply the same as the unknown cases, conditioning on the knowledge that the suspect has a set of particular alleles. Astute readers might also notice that the suspect case should

consider both $[a, b, \ldots]$ and $[b, a, \ldots]$, but that can be shown to be equal to the above.

To further simplify the mathematics, we will focus on improving the likelihood computation of the unknowns-only cases; the reader is encouraged to repeat the analysis and make necessary modifications for the cases involving a suspect.

The naive approach to implementing this model is to directly compute the likelihood using the equations above. However, doing so causes the calculation time to increase dramatically as the number of unknowns increases. To see this, first note that the calculation time is roughly proportional to the number of times $f_{\ell,n}$ is evaluated, which is $|A_\ell|^{2n}$ times. Recall that n is the number of people involved in the hypothesis (and therefore the number of unknowns). If A_ℓ is fairly large, say 15 or so alleles, then each additional unknown increases the calculation time by over 200 times. To put this in perspective, the 3-unknowns hypothesis takes about roughly 20 minutes to an hour. For 4 unknowns, the calculations would take some time between 3 to 9 days.

The naive algorithm is slow, but that is because there are wasted computations. For example, take the 2-unknowns hypothesis. For any different alleles a, b, c, and d of some locus ℓ, the naive algorithm calculates $f_{\ell,2}$ for all permutations of the alleles; that is, it calculates $f_{\ell,2}([a, b, c, d])$, $f_{\ell,2}([a, b, d, c])$, $f_{\ell,2}([d, a, b, c])$, and so on. However, since $f_{\ell,n}$ is not affected by the order of the alleles, this value is equal to the number of permutations of the alleles, multiplied by $f_{\ell,2}([a, b, c, d])$. To utilize this fact in our algorithm, we use the notion of a multiset of X, which is a function over a set X and maps each element of X to a non-negative integer, indicating the number of occurrences, or count, of the element in the multiset. For example, the alleles in the example above can be represented by a multiset m of A_ℓ, where

$$m(x) = \begin{cases} 1, & x = a, b, c, \text{ or } d \\ 0, & \text{otherwise} \end{cases}$$

As an another example, if the alleles were $[a, a, b, c]$, then the multiset would look like

$$m(x) = \begin{cases} 2, & x = a \\ 1, & x = b \text{ or } c \\ 0, & \text{otherwise} \end{cases}$$

We can define the following operations of a multiset m of A_ℓ as follows:

$$|m| = \sum_{a \in A_\ell} m(a) \qquad \text{(size)}$$

$$perm(m) = \frac{|m|!}{\prod_{a \in A_\ell} m(a)!} \qquad \text{(perm. with repeats)}$$

$$a \in m \iff m(a) > 0 \qquad \text{(membership)}$$

where $x!$ denotes the factorial of x. Also, define $M_n(X)$ as the set of all multisets m of X, where $|m| = n$. Define the function s_{A_ℓ}, which takes a multiset m of

A_ℓ, as:

$$s_{A_\ell}(m) = \prod_{a \in A_\ell} p(a)^{m(a)} \begin{cases} 1 - \alpha^{m(a)-1} D_o^{m(a)}, & \text{if } a \in m, a \in CSP_\ell \\ \alpha^{m(a)-1} D_o^{m(a)}, & \text{if } a \in m, a \notin CSP_\ell \\ p(a) D_i, & \text{if } a \notin m, a \in CSP_\ell \\ 1, & \text{if } a \notin m, a \notin CSP_\ell \end{cases}$$

Note that s is similar to f, but not exactly the same. It folds in the probability of having the alleles of m, and removes the no-drop-in factor. Define the function t_ℓ, which takes a multiset m over A_ℓ and returns the multiset where all the counts of alleles that appear in CSP_ℓ are larger by 1. That is, for a particular multiset m,

$$(t_\ell(m))(a) = \begin{cases} m(a) + 1, & a \in CSP_\ell \\ m(a), & \text{otherwise} \end{cases}$$

Finally, we can construct the multiset equivalent equation of the model:

$$n \text{ unknowns} = \left(\sum_{m \in M_{2n}(A_\ell)} perm(m) s_{A_\ell}(m) \right) - \left((1 - \bar{C}) \sum_{m \in M_{2n-|CSP_\ell|}(A_\ell)} perm(m) s_{A_\ell}(t_\ell(m)) \right)$$

which can be verified to be equivalent to the original equation. The equation above may seem more convoluted than the original equation, but in this form, it is possible to take advantage of the structure of the two terms of the subtraction to speed up the computation using dynamic programming.

Let us first consider only the first term and generalize it into a function X, which takes a set of alleles A and a non-negative integer n:

$$X(A, n) = \sum_{m \in M_n(A)} perm(m) s_A(m)$$

It can be shown that, if A_{left} and A_{right} form a nontrivial partition of A (that is, $A_{left} \cup A_{right} = A; A_{left} \neq A; A_{right} \neq A$), then

$$X(A, n) = \sum_{j=0}^{n} \binom{n}{j} X(A_{left}, j) \cdot X(A_{right}, n - j)$$

A similar equation Y can be constructed for the second part of the subtraction. Ideally, the partitions should be of roughly equal size. If in the event that there is only one element in A, then $X(A, n)$ is simply calculated using the first definition.

By calculating X and Y using the recurrence noted above, we can calculate the likelihood using the following:

$$n \text{ unknowns} = X(A_\ell, 2n) - (1 - \bar{C}) Y(A_\ell, 2n - |CSP_\ell|)$$

Thus, the computation time is equal to the time spent calculating X, which is proportional to the number of calls to X, plus the time spent calculating Y, which is proportional to the number of calls to Y. To calculate the number of calculations needed to solve for $X(A, 2n)$, we define a function $T(\eta, \alpha)$ that represents roughly the number of calculations to calculate $X(A, \eta)$ where $|A| = \alpha$. T is defined recursively as follows:

$$T(\eta, \alpha) = \sum_{j=0}^{\eta} \left(T(j, \left\lfloor \frac{\alpha}{2} \right\rfloor) + T(\eta - j, \left\lceil \frac{\alpha}{2} \right\rceil) + 1 \right)$$
$$T(\eta, 1) = 1$$

The solution to the recurrence relation here shows that T is on the order of $\alpha^{1+\frac{\log \eta}{\log 2}}$. Thus, to calculate $X(A, 2n)$, we need on the order of $|A_\ell|^{1+\frac{\log 2n}{\log 2}}$ calculations. A similar result can be discovered for Y. Comparing this to the naive approach, we see that this new algorithm takes less relative time as the number of unknowns increases.

References

[1] Balding, David J., and John Buckleton. "Interpreting low template DNA profiles." *Forensic Science International: Genetics* 4.1 (2009): 1-10.
