# Supplementary material for: Lab Retriever: a software tool for calculating likelihood ratios incorporating a probability of drop-out for forensic DNA profiles
Source: BMC Bioinformatics. 2015 Sep 18;16:298. doi: 10.1186/s12859-015-0740-8 (PMC4575494; doi:10.1186/s12859-015-0740-8)
Supplement: Additional file 2: — Description of the drop-in model used by Lab Retriever. (PDF 90 kb) [file 12859_2015_740_MOESM2_ESM.pdf]

Additional File 2

Description of the drop-in model used by *Lab Retriever*

Here we describe the drop-in model used by *Lab Retriever*. The model was initially described by Balding and Buckleton, however, certain modifications to the model implemented in the R code were made by David Balding. Because these modifications have been implemented in *Lab Retriever*, we will describe the model more fully below.

First, let $P(D_I)$ denote the probability that exactly 1 allele will drop in at a given locus. The probability of 2 alleles dropping in would be $P(D_I)^2$. Assuming a given locus has K alleles, at most K alleles could drop in. The probability of K alleles dropping in would then be $P(D_I)^K$. This is a reasonable model as long as $P(D_I)$ is small and it cannot be >0.5.

We next wish to compute the probability that 0 alleles drop-in. The probability of 0 alleles dropping in will be equal to one minus the probability that 1, 2, 3…or K alleles drop-in. Note that the probability of 1, 2, or 3 alleles dropping in can be written as $P(D_I) + P(D_I)^2 + P(D_I)^3$. This is a geometric sum that can be written for all K alleles as

$$\frac{P(D_I)(1-P(D_I)^K)}{1-P(D_I)}.$$

Thus, the probability of 0 alleles dropping in is simply

$$1-\frac{P(D_I)(1-P(D_I)^K)}{1-P(D_I)}.$$

The above equations are used when computing the numerator and denominator of the LR. In practice, the complete evidence profile is first imported into *Lab Retriever* and appears in the "detected" column (Figure 1). Next, any profiles that are designated as assumed, are imported and appear in the "assumed" column. Alleles in the assumed columns are those from assumed contributors (i.e. contributors that are included in both hypotheses being compared under the LR) or alleles in stutter positions that are above the analytical threshold, but are below the stutter threshold. At these positions, we know that there is a contribution from the assumed contributor, or from stutter. However, it is ambiguous as to whether there is any DNA from an additional contributor. The assumed alleles are completely uninformative on this. The probability of an assumed allele being present, by definition, is 1. Thus, for this reason, alleles from assumed contributors provide no information to the LR calculation regarding the suspected contributor and are not considered in the calculation. Alleles that will be compared to the profile under consideration appear in the "unattributed" column. Finally, during the calculation, alleles found in the evidence profile that remain "unattributed", and that are not accounted for by the suspected contributor are treated as drop-in.

For example, consider the numerator for a single source sample in which the unattributed alleles in the evidence profile are 13 and 14. In this example, the suspected contributor has a 13,13 genotype. Thus, the 14 allele in the evidence profile would be accounted for by drop-in. Here the numerator of the LR would be:

P(Evidence=13,14 | Suspected contributor has 13,13)= *P*(14 drops in| 1 allele drops in)*P*(1 allele drops-in)*P*(No drop-out of 13,13 homozygote).

The quantity *P*(1 allele drops-in) is simply $P(D_I)$. The quantity *P*(14 drops in | 1 allele drops in) is included to account for the fact that even if an allele has dropped-in, we need to consider the probability of it being a 14 allele. We set *P*(14 drops in | 1 allele drops in) equal to the population allele frequency of the 14 allele. Note, as described above, the potential for drop-in is ignored at positions at which we have already designated masking alleles from an assumed contributor; thus masking trumps drop-in for consideration as a factor in the calculation of the LR. Further, we re-scale the population allele frequencies such that all of the frequencies of the alleles in the un-masked positions will sum to one. Specifically, let p_i denote the frequency of the *i*th allele from the population allele frequency database. Next, assume that there are L possible alleles at a given locus that are not in masking positions. Then

$$p_{i,DI} = \frac{p_i}{\sum_{j=1}^{L} p_j},$$

where $p_{i,DI}$ is the probability of allele i dropping-in, given that an allele has dropped in.
